# Strategies for engineering oncolytic viruses to enhance cancer immunotherapy

**DOI:** 10.3389/fphar.2024.1450203

**Published:** 2024-09-06

**Authors:** Ziyang (Steve) Yin, Zhengfeng Wang

**Affiliations:** ^1^ Concordia International School Shanghai, Shanghai, China; ^2^ Department of Hepatobiliary Surgery, The First Affiliated Hospital of Zhengzhou University, Zhengzhou, China

**Keywords:** non-small cell lung cancer, immune checkpoint inhibitors, oncolytic viruses, cancer immunotherapy, tumor microenvironment

## Abstract

Non-small cell lung cancer (NSCLC) is the predominant form of lung cancer and is characterized by rapid metastasis and high mortality, presenting a challenge for early-stage treatment modalities. The heterogeneity of NSCLC’s tumor microenvironment (TME) significantly influences the efficacy of anti-PD-1 immune checkpoint inhibitors (ICIs) therapy, leading to varied patient responses. This review characterized different strains of oncolytic viruses in NSCLC and the different gene edits in pre-existing oncolytic viruses. This study also aimed to provide strategies to enhance anti-PD-1 therapy in NSCLC by engineering oncolytic viruses (OVs). This study offers insights into the genomic adaptations necessary for OVs targeting NSCLC, identify genetic determinants of anti-PD-1 response variability, and propose genomic edits to bolster therapy effectiveness. The primary goal of this study is to present a theoretically designed OV with a detailed genomic framework capable of enhancing the response to anti-PD-1 therapy, thereby advancing the field of cancer immunotherapy.

## 1 Introduction

Lung cancer ranks as the second most commonly diagnosed cancer globally, with 2.2 million new cases reported in 2020, accounting for 11.4% of all new cancer diagnoses. It is also the leading cause of cancer-related deaths, with an estimated 1.8 million fatalities per year (Sung et al., 2021). Lung cancer can be split into two types, small-cell lung cancer (SCLC) and non-small cell lung cancer (NSCLC). SCLC is the more deadly of the two lung cancer types—with a 5-year survival rate of 7%—while NSCLC is less deadly but the more common of the two (85%) (Wang et al., 2020; ACS, 2022). According to NSCLC can be further grouped into lung adenocarcinomas (40%), squamous cell carcinoma (25%–30%), and large cell carcinoma (10%–15%) (UK, 2022).

Surgery, chemotherapy, radiotherapy, immunotherapy, and targeted therapy are the most common approaches to treat lung cancer. The application of these treatments depends on the stage of cancer the tumor is in upon diagnosis. For stage I and II NSCLC patients, surgical resection is the primary and preferable treatment (Howington et al., 2013). Additionally, neoadjuvant and adjuvant chemotherapy has been approved before and following the resection of stage II or III NSCLCs to increase survival rates (Lackey and Donington, 2013; ACS, 2024a). For patients that have a resectable tumor (typically stages 0, I, II, and IIIa), chemotherapy is usually used neoadjuvant or adjuvant to the operation (Chaft et al., 2021; ACS, 2024c). Typically, cisplatin is used for postoperative chemotherapy, significantly reducing the risk of death in NSCLC (Bradbury et al., 2016). Chemotherapy can also be combined with radiotherapy (also called chemoradiation), prescribed for NSCLC patients in stages IIIa and beyond (ACS, 2024c). Chemotherapy can also synergize with immunotherapy (Usually given to NSCLC patients in stage IIIb and above), with chemotherapy killing tumor cells, reducing immunosuppressive substances released by the tumor, and enhancing the anti-tumor response (ACS, 2024c). PD-L1 expression is also increased after treatment by chemotherapy (Chaft et al., 2021). Although chemotherapy might be efficient at killing cancer cells, the treatment has two major downsides: 1) the chemotherapy resistance in cancerous cells leads to decreased efficacy of the treatment, resulting in an incapability of improving patient outcomes; 2) the cytotoxicity of chemotherapies also indiscriminately kills host cells, resulting in many side effects including nausea and loss of hair (ACS, 2024a). Hence, a more specific alternative has been derived, such as targeted therapy. Angiogenesis inhibitors [inhibit the process of angiogenesis and prevent the formation of blood vessels in tumors, diminishing the cancer’s access to nutrients via the bloodstream, leading to slowed cancer growth and a weaker tumor microenvironment (Daum et al., 2021; ACS, 2024b)], epidermal growth factor receptors (ERBBs) inhibitors [block the signal for cancer cell growth since the EGFR mutations are present in about 31.6% of NSCLC cases (Kumari et al., 2019)], and RAS/MAPK signaling inhibitors [block the signal for cancer cell growth and induce cell death, mutations of KRAS/MAPK singling pathway is the main drivers of NSCLC (Xie et al., 2021; Liu et al., 2022)] are commonly used traditional target therapy drugs for NSCLC. Similar to chemotherapy, however, both primary and acquired drug resistance remains a major problem in targeted therapy approaches (Liu et al., 2022).

## 2 Immunotherapy in NSCLC

**FIGURE 1:**
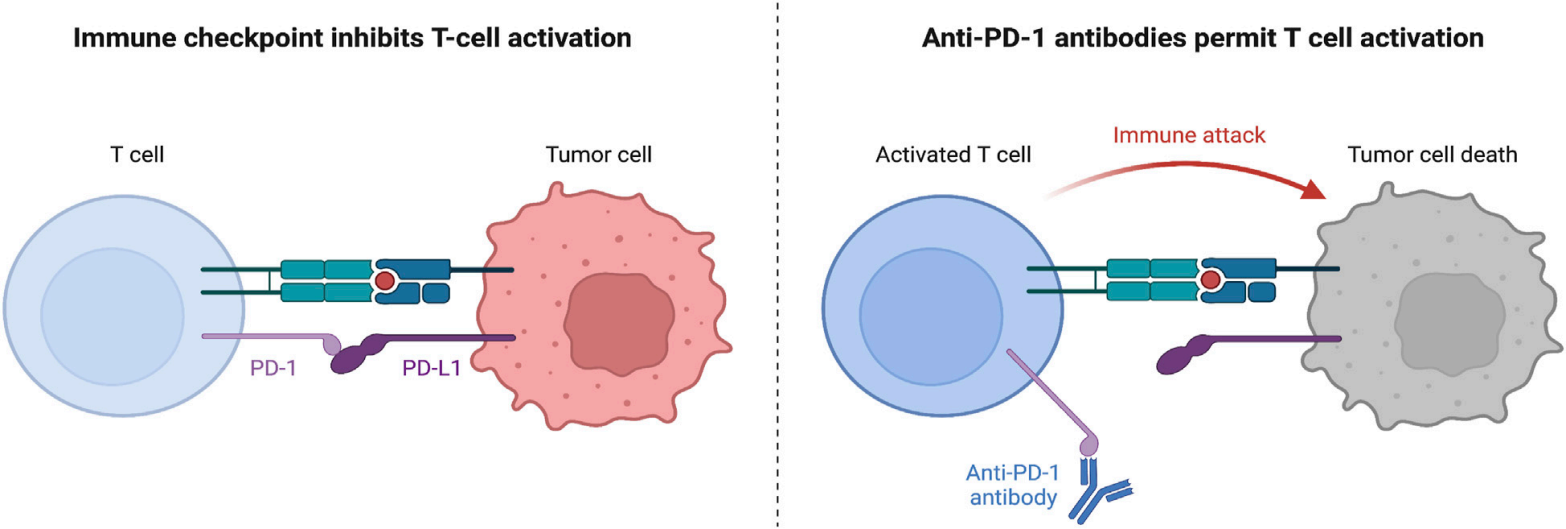
Mechanism of PD-1 and PD-L1 Function. Left: without anti-PD-1 therapy, when CD8^+^ T cells attach their TCR onto the major histocompatibility complex I (MHC I) expressed on the tumor cell, the tumor cell would express PD-L1 attaching to the PD-1 on the CD8^+^ T cell surface and inhibiting T cell activation, proliferation, survival, and cytotoxic secretion (Han et al., 2020). Right: however, with anti-PD-1 therapy, a monoclonal antibody can attach to the PD-1 receptor on the T cell surface, preventing signaling with the PD-L1 ligand, and allowing the T cell to secrete granzymes and perforin to kill the tumor cell (Nie et al., 2021).

Immunotherapy is a form of targeted therapy that particularly stimulates the host’s immune system in order to fend against foreign cancer cells. Generally, this treatment improves the immune system’s capability of antigen recognition or cytotoxicity, leading to longer survival in patients (Chan and Hughes, 2014). Immune checkpoint inhibitor (ICI)-based therapies have been approved for NSCLC. Immune checkpoints (ICs) are steps in the immune response cycle that regulate self-tolerance. ICs attach to co-stimulators of the T-cell receptors on the surface of cytotoxic T cells, signaling T-cell deactivation and death. Normally, ICs are expressed on the surface of host cells; however, on malignant cells, these ICs are upregulated on the surface, resulting in a weaker immune response (NCI, 2022). ICIs are monoclonal antibodies that attach to either the ligand or the receptor of an IC, resulting in the inhibition of the inhibitory signal that deactivates the T-cell response. One common example of an IC complex is the programmed death ligand-1(PD-L1), which is situated on the cancer cell, and the programmed death receptor-1(PD-1), which is situated on the T cell (Alsaab et al., 2017). When PD-L1 attaches to PD-1, the T cell’s cytolytic activity is inhibited and the T cell is deactivated (Alsaab et al., 2017). To prevent this, a monoclonal antibody that binds to either PD-1 or PD-L1 is introduced, preventing the signaling complex to form and allowing the T cell to release cytotoxic damage upon the target cell (Figure 1).

Beyond ICIs, chimeric antigen receptor (CAR) cell therapy is currently being evaluated in many clinical trials for NSCLC. In CAR-T cells, CARs are receptors formed by merging tumor-specific single chain variable fragments (scFv) with the CD3zeta chain of the T cell receptor (TCR) (Morgenstern and Irwin, 2019). Patient T-cells are extracted and encoded to express these CARs according to the specific genetic coding of their cancer. Upon insertion back into the patient, the new CAR T-cells can initiate cytotoxicity through recognition of tumor-antigens, of which the specificity is defined by the scFv (Xiao et al., 2021). While CAR T cell therapy is a promising treatment, especially for hyper-progressive NSCLC patients, CAR therapy is patient specific and hence cannot be mass produced—resulting in high costs—and could cause self-cytotoxicity due to the scFv fragment recognizing host antigens (Liu et al., 2020). Some alternatives have been developed in hopes of mitigating these consequences. One alternative is CAR natural killer (NK) cell therapy, which has the advantage of possessing broader anti-tumor activity and targeting even MHC downregulated targets, being non-patient specific giving potential to allogeneic CAR NK cell therapy, and less cytokine stimulation decreasing the chances of cytokine release syndrome (Albinger et al., 2021). Another alternative is bi-specific T cell engager (BiTE) therapy, which is made of two antibodies, one targeting tumor-associated antigens and another targeting TCRs, connected through a short constant region in the middle, drawing the tumor cell closer to T cells, increasing antigen recognition and immune response (Goebeler and Bargou, 2020). Recent studies have shown that CAR T-cells can be edited to secrete BiTEs (called CAR.BiTE cells) with the potential of mitigating antigen escape without increasing the toxicity of CAR T cell therapy (Choi et al., 2019).

### 2.1 Flaw of immunotherapy

However, despite the benefits, immunotherapy has an Achilles heel: tumor cells are able to develop resistance. Less than 30% of NSCLC patients response to current immunotherapies. The three known classifications of immunotherapy resistance in cancers are shown in Table 1 (Sharma et al., 2017; Zhou and Yang, 2023). The progression of resistance is accelerated in NSCLC due to its fast growth rate increasing replicative stress and instances of replicative error—both of which contribute to tumor heterogeneity and genome mutation in immunotherapy resistance (Wang et al., 2012; Zhou and Yang, 2023). Biomarkers that help indicate resistance and immunotherapy efficacy in NSCLC are included in Table 2. Many means of surmounting immunotherapy resistance have been invented, the common ones include: 1) targeting co-inhibitory signals such as the ICIs PD-1, CTLA-4, LAG-3, TIM-3, TIGIT, VISTA, and Siglec-15 to decrease inhibition of immune response (Frisone et al., 2022); 2) enhancing co-stimulatory signals to improve the immune response of cytotoxic cells such as T and NK cells (For patients of NSCLC, the use of CD134 (OX-40), CD137(4-1BB), and IL15 are currently under investigation (Croft, 2009; Frisone et al., 2022); 3) priming the immune system to tumor neoantigens with vaccine therapy to increase antigen recognition and effector functions of T and B cells (Makker et al., 2022); and 4) combining immunotherapy with other therapies such as chemotherapy increases response due to increased neo-antigen release as a result of chemo-induced cell death (Rheinheimer et al., 2020; Frisone et al., 2022). There are many different methods in clinical trial attempting to remedy these resistances in NSCLC, these methods include: targeting co-inhibitory signals such as ICIs and enhancing co-stimulatory signals such as CD134 to increase immune activation, increasing tumor sensitivity through vaccine therapy to increase antigen recognition, and through pre-treatment of other co-therapies such as chemotherapy and radiotherapy (Frisone et al., 2022; Zhou and Yang, 2023). Additionally, nanotechnology has become popular in the search to overcome immunotherapy resistance. Not only can nanoparticles promote T-cell enrichment and activation by using magnetic fields to promote TCR aggregation, they can also improve the bioavailability of insoluble drugs and prolong drug circulation by making the treatments more stable (Shao et al., 2023; Zhang et al., 2023). One specific example are albumin-based nanoparticles that can be encased on proteins to bypass drug-prevention complexes such as drug efflux (Hassanin and Elzoghby, 2020). Zhejiang University had conducted a clinical study to determine the safety and efficacy of platinum-based albumin-bound paclitaxel regimen in the treatment of stage IIB and IIIA NSCLC (Zhao, 2015). However, even with these methods of overcoming resistance, immunotherapeutic treatments fail to treat cancer with consistency and efficacy (Frisone et al., 2022). Hence, in this paper, an additional method of immunotherapy—an oncolytic virus-based cancer vaccine—is proposed.

**TABLE 1:** Types and mechanisms of resistance to immunotherapy.

Type	Description	Mechanisms
Primary Resistance	Cancer is not recognized and hence does not respond to the immunotherapy	Tumor cell intrinsic - Lack of antigens - Lack of antigen presentation - T cell exhaustion
Adaptive Resistance	Cancer is recognized by the immune system but adapts and evades the immunotherapy	Tumor cell extrinsic - absence of T cells - immune checkpoints - immunosuppressive cells
Acquired Resistance	Cancer initially responds to immunotherapy, but resistance builds up after a period of time	- Tumor heterogeneity - Mutation of tumor genome

**TABLE 2:** Biomarkers for immunotherapy sensitivity.

Biomarker	Type	Correlation	References
TIL quantity	TME	Positive	Dong et al. (2016), Zeng et al. (2016)
CD8A and CD274 expression	TME	Positive	Geng et al. (2015), Fumet et al. (2018)
*Clostridia a*bundance in gut	Gut Microbiome	Positive	Sivan et al. (2015), Wargo et al. (2017)
Ruminococcaceae family abundance in gut	Gut Microbiome	Positive	Gopalakrishnan et al. (2018)
*Agathobacter muciniphila* abundance in gut	Gut Microbiome	Positive	Routy et al. (2018)
*Bacteroidales* abundance in gut	Gut Microbiome	Negative	Sivan et al. (2015), Wargo et al. (2017)
EGFR mutation	Genome Mutations	Negative	Cabezón-Gutiérrez et al. (2021), Shi et al. (2022)
STK11/LKB1 mutation	Genome Mutations	Negative	Biton et al. (2018), Skoulidis et al. (2018)
JAK1/2 mutation	Genome Mutations	Negative	Garcia-Diaz et al. (2019)
PTEN deletion	Genome Mutations	Negative	Peng et al. (2016)
MDM2 amplicfication	Genome Mutations	Negative	Oliner et al. (2016), Adashek et al. (2020)
CDKN2A/B deletion	Genome Mutations	Negative	Champiat et al. (2017), Kato et al. (2017)
DNMT3A mutation	Genome Mutations	Negative	Kato et al. (2017), Adashek et al. (2020)

## 3 Oncolytic viruses

Oncolytic viruses (OVs) are modified viruses that specifically target tumors, carry and induce the expression of transgenes, and cause direct tumor cell lysis (NCI, 2018). These viruses can be edited to perform certain functions against specific cell types (Kaufman et al., 2015). There are multiple types of oncolytic viruses, each with its unique receptors, genome size, effectiveness, and mechanisms of action (Rahman and McFadden, 2021). Table 3 lists OVs that have shown promise in treating lung cancers. OVs has shown promising results in cancer treatment and have been widely used for cancer treatment, including advanced-stage melanoma and glioblastoma (GBM). Currently, several viruses, including vaccinia virus, coxsackievirus, adenovirus, reovirus, and herpes simplex virus have been tested in NSCLC. The different ongoing clinical trials for oncolytic viruses targeting NSCLC are listed in Table 4.

**TABLE 3:** Examples of oncolytic viruses.

Types	OV	Genome size	Receptors	Example
DNA Virus	Adenovirus (AdV)	35 kb (dsDNA)	CAR, DM2, CD80 (B7-1), CD86 (B7-2), CD46	DNX-2401, ONCOS-102 (Cai et al., 2017; Hensen et al., 2020)
	Herpesvirus (HSV)	154 kb (dsDNA)	HVEM, Nectin 1, Nectin 2	T-VEC, HSVG207, M032 (Ma et al., 2018)
	Parvovirus	5 kb (ssDNA)	Sialic acid residues, P antigens	ParvOryx 01(Kulkarni et al., 2021)
	Poxvirus	130–360 kb (dsDNA)	Heparan, laminin, CD98, chondroitin, integrin β1	JX-594, Pexa-Vec (Kawai et al., 1988; Kirn and Thorne, 2009; Chan and McFadden, 2014; MacLeod et al., 2015; Jayawardena et al., 2020)
	Vaccinia virus	190 kb (dsDNA)	MARCO, Glycosaminoglycans	
RNA Virus	Coxsackievirus	28 kb (SS + RNA)	CAR, ICAM-1, DAF	CVA21, CV-B3 (Geisler et al., 2021)
	Measles Virus	16 kb (SS- RNA)	CD150 (SLAMF1), CD46, Nectin 4	MV-NIS (Engeland and Ungerechts, 2021)
	Newcastle disease virus (NDV)	15 kb (SS- RNA)	Sialic acid	MEDI5395 (H. Song et al., 2019; Ye et al., 2018)
	Reovirus	23 kb (dsRNA)	Sialic acid, JAM1	Reolysin (Müller et al., 2020)

**TABLE 4:** Clinical trials involving NSCLC associated OVs.

Oncolytic virus	Responsible party	Description	Start date	Status
MEM-288	Memgen, Inc	Dose-escalation of MEM-288 monotherapy in solid tumors (includes NSCLC) to determeine maximum tolerated dosage (MTD) and reccomended phase II dose (RP2D)	2022-02-03	On-going
R130	Shanghai Yunying Medical Technology	A clinical safety and efficacy study on R130 injection for treatment of relapsed/refractory advanced solid tumors (includes NSCLC)	2023-03-30	On-going
T3011	ImmVira Pharma Co. Ltd	Saftey and tolerability check of OV T3011 given via intratumoral injection in advanced solid tumors (includes NSCLC)	2020-04-21	On-going
ADV/HSV-tk	The Methodist Hospital Research Institute	Determine efficiacy and safety of stereotactic body radiation therapy and *in situ* OVT (an adenovirus-mediated expression of herpes simplex virus thymidine kinase) as pre-therapy for pembrolizumab in NSCLC and triple negative breast cancer	2017-07-01	Completed
TG6050	Transgene	Determine optimal dose and schedule of administion of OV TG6050 in advanced NSCLC patients	2023-04-05	On-going
	Transgene	Dose-escalation study of cotherapy of intra-tumoral BT-001 (TG6050) injections and intravenous pembrolizumab in advanced solid tumors (includes NSCLC)	2021-02-25	On-going
Ad-MAGEA3 MGA1-MAGEA3	Turnstone Biologics, Corp	Dose-escalation trial of Ad-MAGEA3 and MGA1-MAGEA3 in combination with pembrolizumab in NSCLC patients who have had at least one cycle of platinum based chemotherapy or one treatment of anti-PD-1/PD-L1 therapy	2017-03-08	Completed
RP1	Replimune	Dose-esclation trial of RP1 monotherapy and RP1 nivolumab cotherapy in advanced or refractory tumors (includes NSCLC) to determine MTD.	2017-09-20	On-going
VSV-IFNβ-NIS	Vyriad, Inc.	Identify optimal dosage and assess efficacy of VSV-IFNβ-NIS with pembrolizumab in patients with solid tumors. (includes NSCLC)	2019-04-09	On-going
ColoAd1	Akamis Bio, Inc.	Assess pattern of ColoAd1 delivery and viral expression when administered by intra-tumoral injections in various cancers (includes NSCLC)	2015–04	Completed

### 3.1 Commonly used oncolytic viruses

Vaccinia Virus (VV): VVs are large, enveloped, double-stranded DNA (dsDNA) viruses belonging to the poxvirus family, each containing approximately 190 kbps, encoding around 250 genes. The Lister, Wyeth, and Western Reserve strains of VV are more commonly used in OV research as they can incorporate large amounts of foreign DNA without affecting the virus’s replicative efficacy (Guo et al., 2019; Guse et al., 2011). VVs do not have a specific receptor making them a prominent candidate for multiple types of cancers (Guo et al., 2019; Guse et al., 2011). However, in the interest of lung cancer, VVs preferably target cells with expression of the scavenger receptor MARCO, which, according to the human protein atlas (HPA), is enhanced in lung cancers (Sjöstedt et al., 2020). Additionally, VVs have innate specificity for cancer cells due to their sensitivity toward type-1 IFNs (Guo et al., 2019; Guse et al., 2011). Furthermore, VVs can modulate the TME and evade immunosuppression by secreting virokines (viral proteins that resemble cytokines and chemokines), and viroceptors (viral proteins that act as decoy receptors for cytokines and chemokines), evading immunosuppression within the TME and prolonging viral infection and replication (Truong and Yoo, 2022). VV virion entry is also accelerated by low pH environments due to their low-pH-dependent endosomal pathway, hence making them particularly effective in the TME (Townsley et al., 2006). Additionally, one final feat of VVs is its capabilities to replicate independent of the host cell’s genome due to having its own RNA transcriptase and transcription factors within its viral core (Tolonen et al., 2001). This allows the VV to be even more flexible with its surrounding conditions whilst conducting pathogenesis.

Herpes simplex virus (HSV): HSVs are large, enveloped, dsDNA viruses belonging to the herpesviral family, containing around 152 kbps (Aldrak et al., 2021). The main receptor of HSV is Nectin-1, which according to the HPA, is overexpressed in lung cancers, albeit inconsistently (Farooq et al., 2010; Sjöstedt et al., 2020). Additionally, an alternative receptor for HSV, HVEM, is found to be overexpressed in patients of non-small cell lung cancer with N2 metastasis or later, making HSV relatively specific for lung cancer (Farooq et al., 2010; Ren et al., 2018). Similar to VVs, HSVs have a wide selection of genes that can not only help them navigate the TME and perform immunoevasion but also be manipulated to increase specificity and efficacy (Hong et al., 2022).

Adenovirus Serotype 3 (AdV3): Adenoviruses are a family of viruses capable of holding 25–45 kbps of dsDNA. Many different serotypes of AdVs have been identified, however, the receptors of AdV serotype 3 (AdV3) are more specific toward lung cancer (Zhao et al., 2021). One of the main receptors for AdV3 is desmoglein-2 (DM2), a cadherin that is overexpressed in lung cancers (Wang et al., 2011; Cai et al., 2017; Zhao et al., 2021). Similarly, CD46 is a receptor for AdV3 of which its overexpression is associated with malignant transformation and metastasizing potential (Elvington et al., 2020; Zhao et al., 2021). Co-receptors CD80 (B7-1) and CD86 (B7-2) that are expressed on antigen-presenting cells (APCs) are also receptors of AdV3, allowing for the possibility of AdV3s to infect APCs and provide the tumor-associated antigens directly (if modified to do so) (Zhang and Bergelson, 2005; Zhao et al., 2021). Although AdV3 is very specific for lung cancers, the safety of the virus can be improved by moving the AdV3 fiber knob (a protein that attaches to the virus’s receptor) onto AdV5, replacing the AdV5 fiber knob (5/3 chimerism), taking advantage of the specificity of AdV3 while having the potent but safe lytic activity of AdV5 (Hemminki et al., 2011; Koodie et al., 2019; Zhao et al., 2021).

### 3.2 Delivery routes and therapeutic effects of OVs

Systemic or local administration of the OVs is another contention of discussion, with local administration limiting the virus to one solid tumor and systemic administration raising many other problems. Currently, intratumoral delivery is the most common route of administration for OVs, being more capable of being controlled, and showing more definite therapeutic effects (Li et al., 2020). Although theoretically, viruses can sustain virion production indefinitely within cancer cells until the cancer is eviscerated, however in practice, multiple doses of the OV are needed to even show tumor regression (Ferguson et al., 2012). Regarding systemic delivery of OVs, the method of administration would be intravenous injection, the limitations of such are as follows: 1) neutralization via immune response; 2) non-specific uptake via non-target tissues; 3) indiscriminate cytotoxic damage (Atasheva and Shayakhmetov, 2021). However, despite these limitations, clinical trials of intravenous delivery of OVs have occasionally shown success, an example of which was the intravenous injection of T-VEC in co-therapy with ipilimumab successfully treating stage IIIb-IV melanoma (Puzanov et al., 2016). Alternatively, there are also additional methods of administration that can be performed according to the type of cancer within the patient. Intraperitoneal injections target organs within the abdominal cavity, intrathecal injections target tumors in the central nervous system, and subcutaneous injections target melanomas and soft tissue sarcomas (Chen et al., 2017; Cohn et al., 2017; Li et al., 2020).

**FIGURE 2:**
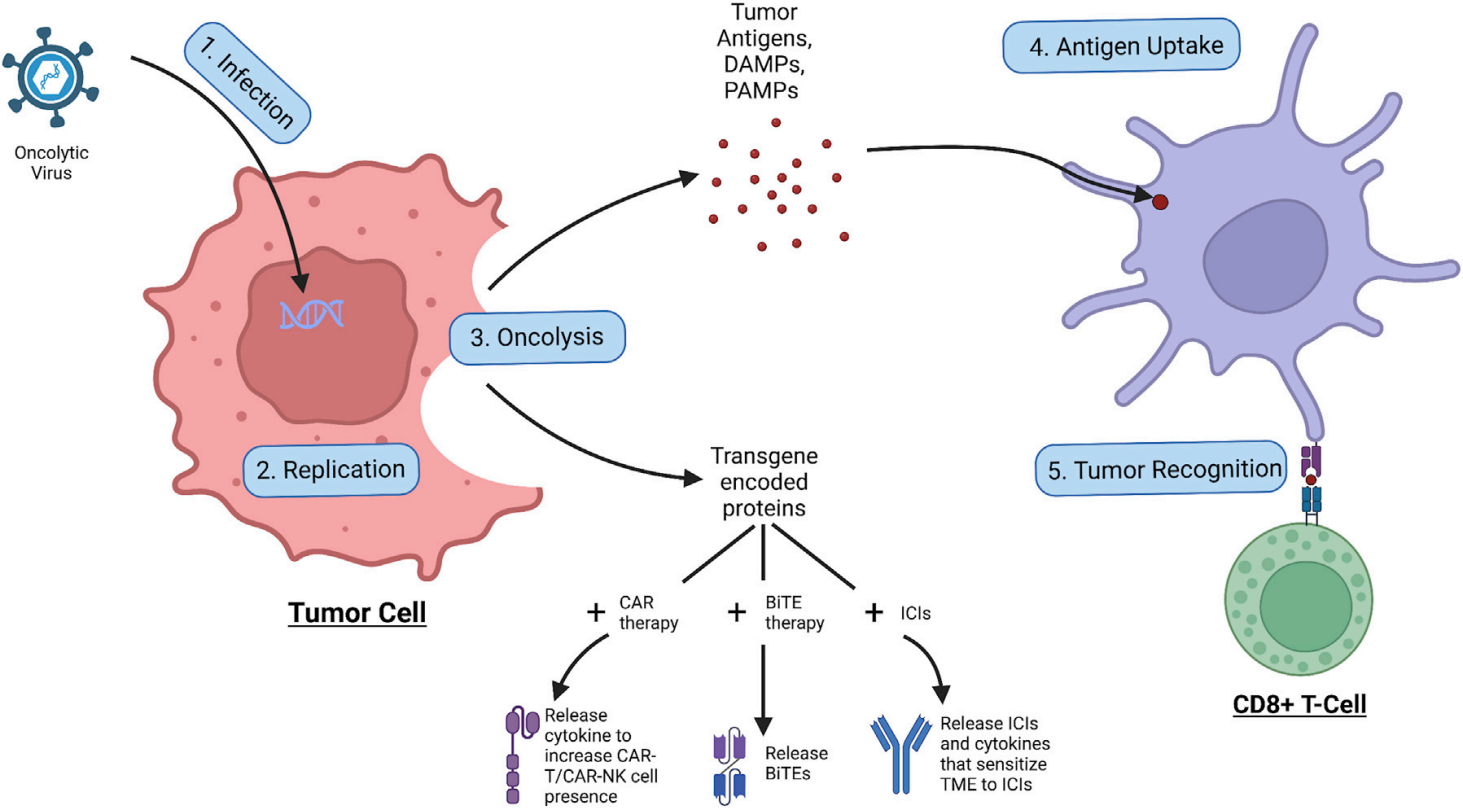
Process of oncolytic virus infection. The OV first infects the tumor cell, allowing for replication of the viral genome, leading to the production of virions and transgene-encoded proteins. Following the OV induces oncolysis, leading to the release of tumor antigens, DAMPs, PAMPs, and transgene-encoded proteins. The tumor antigens are taken up by APCs which use them to activate immune cells such as the CD8^+^ cytotoxic T cell which go to attack cancer. The released transgene-encoded proteins, depending on how they are encoded, can be cytokines that stimulate CAR-T/CAR-NK cell proliferation and migration, BiTEs that are released upon oncolysis, or ICIs and cytokines that sensitize the TME to the ICI effects.

As shown in Figure 2, the OVs first infect the tumor cell through its specific receptor, and subsequently insert its viral genome into the cell which enters the nucleus where the viral genome is replicated, creating virions and the transgene-encoded proteins (Shi et al., 2020). Following this, the OV induces cell lysis of the infected tumor cell and releases tumor antigens, DAMPs, PAMPs, and transgene-encoded proteins. The tumor antigens and DAMPs and PAMPs all result in the increase of immune response to the tumor, with the tumor antigen specifically being uptaken by APCs and presented to immune cells such as the CD8^+^ cytotoxic T-Cell, increasing tumor recognition and intuitively the anti-tumor immune response (Shi et al., 2020). The transgene-encoded proteins on the other hand can be modified to be combined with co-therapies that can further increase tumor regression (Harrington et al., 2019). To improve CAR therapy performance, the transgenes within the OV can be encoded to express cytokines that increase proliferation and migration and prevent inhibition of the CAR-attached immune cell to increase tumor trafficking and regression (Moon et al., 2018). With recent research, the possibility of having CAR T cells as carriers of small doses of OVs to the tumor site could have promising results, however, more research will need to be conducted before a conclusion can be made (VanSeggelen et al., 2015). Similarly, the transgenes can be encoded with BiTEs, allowing BiTE release upon tumor cell oncolysis, which has been shown to have greater therapeutic effects than BiTE therapy alone (Fajardo et al., 2017). Finally, in combination with ICIs, antibodies targeting against immune checkpoints can be encoded within the transgenes, allowing immune checkpoint inhibition upon the oncolysis of the tumor cell (LaRocca and Warner, 2018). The OV transgenes can also contain other proteins that can help sensitize the tumor environment to ICI therapy, such as increasing immune cell presence in the area or reducing the effects of the TME (LaRocca and Warner, 2018). However, it should be noted that the activation of the immune system could also induce antiviral responses, which could lead to the deactivation of viral replication (Harrington et al., 2019). In summary, with the unique aspects of OVs—tumor specificity, transgene expression, and induction of oncolysis—these special viruses can kill tumor cells, release tumor antigens, increase the effects of combinational therapies, and act as a tumor vaccine for the immune system.

One example of a clinically approved oncolytic virus is Talimogene Laherparepvec (T-VEC), a modified oncolytic herpes simplex virus that can be used against melanomas and has been recently authorized for glioblastomas (Ando et al., 2008; Ferrucci et al., 2021). T-VEC has four major edits in its genome: 1) Deletion of ICP34.5: Infected cell protein 34.5 (ICP34.5) activates protein phosphatase-1 (PP1) which dephosphorylates elF2alpha, preventing the inhibition of viral replication (Li et al., 2011; Ferrucci et al., 2021). But with the gene deleted, the protein will no longer be produced, meaning the oncolytic HSV will not be able to inhibit elF2alpha phosphorylation, and hence viral replication will come to a halt. However, in tumor cells, due to their highly replicative nature, the phosphorylation of elF2alpha is constantly inhibited, and hence even without ICP34.5, T-VEC is able to replicate in tumor cells (Liu et al., 2003; Kazemi et al., 2004; Guo et al., 2017). This increases the replicative specificity of T-VEC; 2) deletion of ICP47: ICP47 binds to TAP1 and TAP2 residing on the endoplasmic reticulum (ER) membrane, blocking and preventing peptide transport into the ER lumen, resulting in no antigen presentation by MHC I (Goldsmith et al., 1998). Hence, deleting this gene will permit and increase antigen presentation, which can increase antigen recognition and anti-tumor responses (Ferrucci et al., 2021); 3) early Expression of US11: Unique short glycoprotein 11 (US11) blocks the phosphorylation of elF2alpha by binding to PKR (Cassady et al., 1998), The early expression of US11 allows for the promotion of proliferation of virus within tumor cells while not impairing tumor selectivity (Ferrucci et al., 2021); and 4) Insertion of GM-CSF: The granulocyte-macrophage colony-stimulating factor (GM-CSF) is a cytokine that, when secreted, promotes the myeloid cell development and maturation and dendritic cell differentiation and survival (Egea et al., 2010). Hence, when inserted within the transgenes, the GM-CSFs will promote tumor infiltration (Kumar et al., 2022). However, too much GM-CSF has also been shown to exhaust immune cells and promote cancer growth (Kumar et al., 2022).

### 3.3 OVT co-therapies

Co-therapies are commonly used in conjunction with OVT to increase the efficacy of treatment. One route of co-therapy of OVT is to combine with chemotherapy. Chemotherapy is able to systemically alter the behavior of the tumor and the TME, making it equipped to acts a pre-therapy to OVT (Nguyên et al., 2008). Generally, chemotherapy is able to suppress the immune-surveillance and decrease Treg cell presence (such as MSDC) increasing viral infection rates and decreasing tumor promoting factors respectively. Table 5 lists combinations of chemotherapy and OVT with more specific known synergetic effects summarized from related studies (Nguyên et al., 2008; Heo et al., 2011; Tusell Wennier et al., 2012; Mirbahari et al., 2024). However, the pathways taken in viral replication are very similar to that of those taken by cancer cells. Hence, the synergetic potential of chemotherapy with OVT is limited due to the former inhibiting various functions of the latter in therapy (Nguyên et al., 2008). Besides chemotherapy, OVT can also be in co-therapy with radiotherapy, a combination that has yielded more successful therapeutic results than any of the two alone in many studies (Mansfield et al., 2013; Dai et al., 2014). In Wilkinson et al. (2016) study it was found that not only was vaccinia virus’s viral DNA immune to radiotherapy damage, but there was a slight increase in cytotoxicity when combined. In another study, the increase in cytotoxicity during OV and radiotherapy co-therapy was attributed to the increased release of DAMPs and increased ratio of M1 to M2 macrophages (Chen et al., 2021). Finally, OVT can be in co-therapy with other immunotherapies such as ICIs, angiogenesis inhibitors, CAR therapy, etc (Nguyên et al., 2008). OVT in these instances can act as pre-treatment by either increasing the receptiveness or sensitivity of the environment to the following immunotherapy (Mirbahari et al., 2024). For example, OVs can be deployed first to increase tumor-antigen presence within the TME to increase subsequent CAR T-Cell therapy efficacy due to encouraging antigen uptake and antigen recognition (Shi et al., 2020). This article will focus on the co-therapy of OVs with the ICIs anti-PD-L1/PD-1; this will be expanded more upon in latter sections.

**TABLE 5:** Synergies between OVT and chemotherapy.

Chemotherapy	Synergetic effect	Corresponding Virus(es)
Taxenes	Block cell cycle at the G2/M phase, the phase at which the vaccinia virus is most adapated to infect	Vaccinia Viruses
	Additionally, if an oncolytic vaccinia virus is used as pre-therapyThe consequential release of IFNs after infection and HMGB1 after viral-mediated cell death can sensitize cells to taxenes (Huang et al., 2011)	AdenovirusesHerpesviruses (with ICP34.5 deletion)
		Vaccinia Virus
Cyclophasphomide	Suppress host adaptive immune system, prolonging period of viral replication	All
	Sensitize tumor cells to CD8^+^ T cell mediated apoptosis	
5-Fu		All
	Increase immune antigen expression	All
HDACi	Upregulation of CAR genes and cellular replication rates	Adenoviruses
		Herpesviruses
Rampamycin	Inhibition of IFN production leading to enhanced viral replicative
		RhabdovirusesHerpesvirusesPoxvirusesAdenoviruses
Cisplastin	Upregulation of GADD34 improves viral repliation efficacy	Herpesviruses (with ICP34.5 deletion)
Gemcitabine	Deplete MSDCs and increase immune response efficacy	All
Sunitinib		
Docetaxel		
Retinoic Acid		

## 4 Oncolytic virus engineering

The development of safe, cancer-selective, and efficient OVs against various tumors relies heavily on the genetic engineering of OVs. Both natural and genetically modified viruses have shown promising results in treating various cancers. Understanding the biology and genetics of the virus, the interactions between the virus and the host immunity, and how cancer cells protect themselves from immune cells is critical for the genetic modification of OVs. In general, the aim of engineering OVs is to enable the application of OVs in cancer immunotherapy to broadly activate anti-tumor immune responses, enhance the tumor cell tropism of OVs, and reduce toxicity to normal cells.

### 4.1 Viral genome edits to increase specificity

While OVs have innate specificity towards cancer cells, without moderation, the OVs can still indiscriminately infect host cells. This is because while some receptors may be overexpressed in cancers, a portion of host cells can still express said receptor and would therefore be susceptible to viral infection of the OV. To mitigate this problem, edits to the viral genome are made, these edits can be generalized into the following groups: a) deactivation of viral replication: In most instances, viruses require the production of specific proteins to disrupt the infected cell’s antiviral pathways to prevent the inhibition of viral replication (Magden et al., 2005; Majdoul and Compton, 2022). At the same time, most, if not all, cancers have a faulty replication pathway that is constantly active resulting in uncontrolled tumor growth (Vassilev and DePamphilis, 2017; Yaacov et al., 2021). Hence, in certain instances, if the virus’s mechanism to induce viral replication is deactivated, the virus will no longer be able to reproduce in regular host cells; However, due to the faulty replicative pathway of cancers, the virus would still be able to reproduce in tumor cells (Everts and van der Poel, 2005). This intuitively increases the specificity of the modified viruses towards cancerous cells via exploiting the cancer cell’s constantly active replicative pathway; b) deactivation of viral immuno-evasion: Viruses have pathways of evading the human immune response, an example of this is the B18R protein of vaccinia viruses which acts as a decoy receptor for interferon-α (IFN-α), preventing the IFN-α signaling cascade from inhibiting viral functions (Kim et al., 2017). At the same time, immune signaling proteins such as interferons (IFNs) and cytokines are downregulated and inhibited in the TME (Fenton et al., 2021). Hence, if the immune evading pathways of the viruses were to be deactivated, the virus may not be able to infect and proliferate in normal host cells, whilst they can function normally in the TME. This increases the specificity of the modified viruses towards cancerous cells via exploiting the TME’s immunosuppressive effects; and c) using tumor-specific promoters: Promoters are sequences of DNA that need to be activated by specific proteins (e.g., growth factors) to initiate transcription of downstream genes (NIH, 2024). Tumor cells can have special proteins that can read promoter sequences unique, or uncommon outside of, tumor cells, creating specificity with promoters (Wang et al., 2018). Hence, if certain genes are placed downstream of unique promoters (such as the human telomerase reverse transcriptase (hTERT) promoter), even if an OV infects a normal host cell, the genetic content downstream of the promoter will not be transcribed within the cell, and hence, will not be expressed. The potential viral genome edits for increasing the specificities of different OVs are listed in Table 6.

**TABLE 6:** Choice of viral genome edits.

Virus	Edit	Rationale
Vaccinia Virus (VV)	Delete Thymidine Kinase (TK)	Increase specificity of the OV by limiting viral replication to tumor cells where the salvage pathway of DNA synthesis is more active than in normal host cells
	Delete Vaccinia Growth Factor (VGF)	Increase specificity of the OV by limiting viral replication to tumor cells where the rates of DNA replication and transcription are faster than normal cells
	Delete B18R	Increase specificity of the OV by preventing the secretion of a PKR/elF2alpha pathway inhibiting protein, limiting replication of virus to tumor cells where the pathway is innately deactivated
Herpes Simplex Virus (HSV)	Delete ICP34.5	Increase specificity of the OV by preventing the inhibition of the PKR/elF2alpha pathway, limiting replication of virus to tumor cells where the pathway is innately deactivated
	Delete ICP47	Increase antigen presentation by preventing the inhibition of peptide transport from the cytoplasm into the ER lumen, allowing for increased antigen recognition
	Early Expression of US11	Increase viral replication by expressing viral replication inducing protein US11 earlier, resulting in greater efficacy of the OV.
	Insert GM-CSF	Increase immune cell presence to increase tumor infiltration and antigen recognition
Adenovirus Serovirus 3 (Ad3)	Delete E1A	Increase specificity of the OV by preventing the inhibition of E2F pathway cell cycle shutdown, resulting in the virus only being able to replicate in tumors where the E2F protein is naturally inhibited
	Use of hTERT promoter	Increase specificity of the OV by limiting the transcription of viral genome only cells that have the hTERT binding RNA polymerase, which is used in tumor cells and only a select few of host stem cells

### 4.2 Viral genome edits to increase anti-tumor immunity

Tumor cells often exhibit properties such as evasion of immune surveillance and loss of immunological response. Selected genome edits in OVs would have a scientifically explainable positive impact on anti-tumor immune response. Impacts of edits in OVs were judged to be positive or not based on if they met the following criteria: a) increased replication: Increasing the replication of the virus allows the virus to duplicate faster and intuitively infect more cancer cells leading to greater efficacy. Similarly, an increased replication allows for the genes encoded in the viral genome to be expressed more, also increasing the efficacy of treatment. This increased replicative speed can be achieved via the insertion of enhancers or moving certain viral genes for early expression; b) increased antigen recognition: Antigen recognition is a vital step in the process of an immune response, allowing for the engagement of T and B cell effector function (Apavaloaei et al., 2020). One method of increasing recognition of tumor-specific antigens is the deletion of antigen presentation inhibiting pathways in viruses (e.g., the deletion of ICP47 in Herpes Simplex Virus, which prevents the inhibition of antigen transport through TAP1 and TAP2 into ER lumen for attachment to MHC I) (Ren et al., 2018). Another method of increasing recognition of tumor-specific antigens is the insertion of specific cellular markers that would be unique to the tumor upon infection. This would result in the release of these tumor-specific markers upon tumor cell lysis, increasing the number of tumor antigens, and causing T-cells to recognize and kill the OV-infected tumor cells that are also expressing this marker; and c) increased immune cell infiltration: Increasing the presence of immune cells in an area would not only increase the antigen presentation present, but also increase the number of T cells in the area. This would increase the amount of T cell-tumor cell complexes, increasing the efficacy of anti-PD-1 therapy. One method of increasing immune cell concentration would be to release chemokines and cytokines in the interest of increasing the immune cell population near the infected tumor, increasing the migration of APCs and immune cells, subsequently increasing the chances of antigen presentation (Melcher et al., 2021).

### 4.3 Viral genome edits to increase anti-PD-1 efficacy in NSCLC

As mentioned previously, anti-PD-1 ICI therapy is one form of immunotherapy that has shown great therapeutic results for many cancers; however, ICI therapy has a couple drawbacks that limit its potential effect in patients. Hence, this study aims to design OVs that can enhance and support anti-PD-1 effectiveness for NSCLC patients.

**FIGURE 3:**
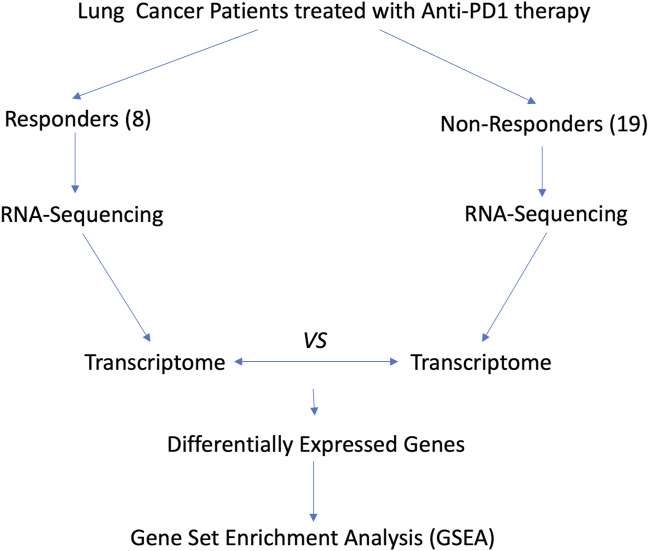
Flow chart for revealing potential targets for designing OVs.

**FIGURE 4:**
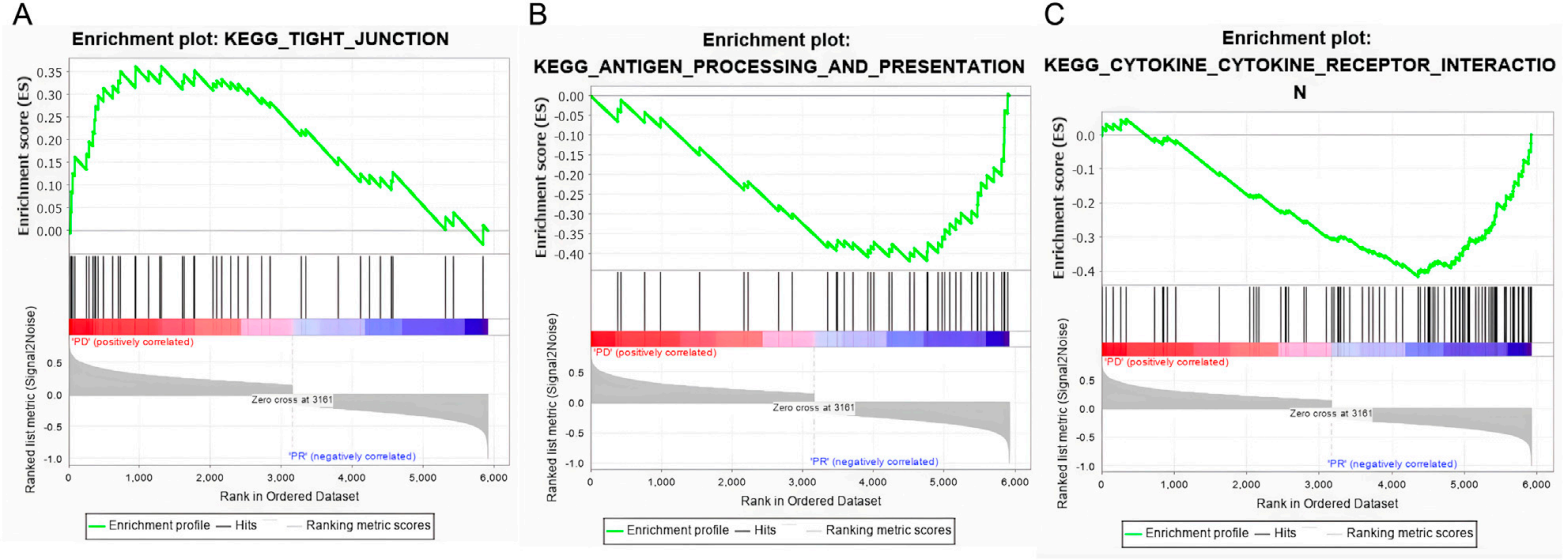
Gene set enrichment plots of three highly correlated pathways. **(A)** The tight junction pathway shows a positive correlation anti-PD1 non-responsive patients (PD). **(B)** The antigen process and presenting pathway is positively correlated with responsive patients (PR). **(C)** The cytokine and cytokine receptor interaction pathway is positively correlated with the PR patients.

As shown in Figure 3, we analyzed the transcriptomes of NSCLC tumor from 27 patients treated with anti-PD-1 therapy. The 27 patients’ transcriptomes have been grouped into eight responders and 19 non-responders. Gene set enrichment analysis (GSEA) was used to compare the transcriptome data between the responders and non-responders, distinguishing which genes are comparatively altered between the two conditions, providing potential targets for OV design. According to the enrichment plots given by the GSEA, several genes that have shown strong correlation with either the PR or PD groups from the pathways shown in Figures 4A–C have been chosen as targets for editing.

#### 4.3.1 Expression of CLDN1

**FIGURE 5:**
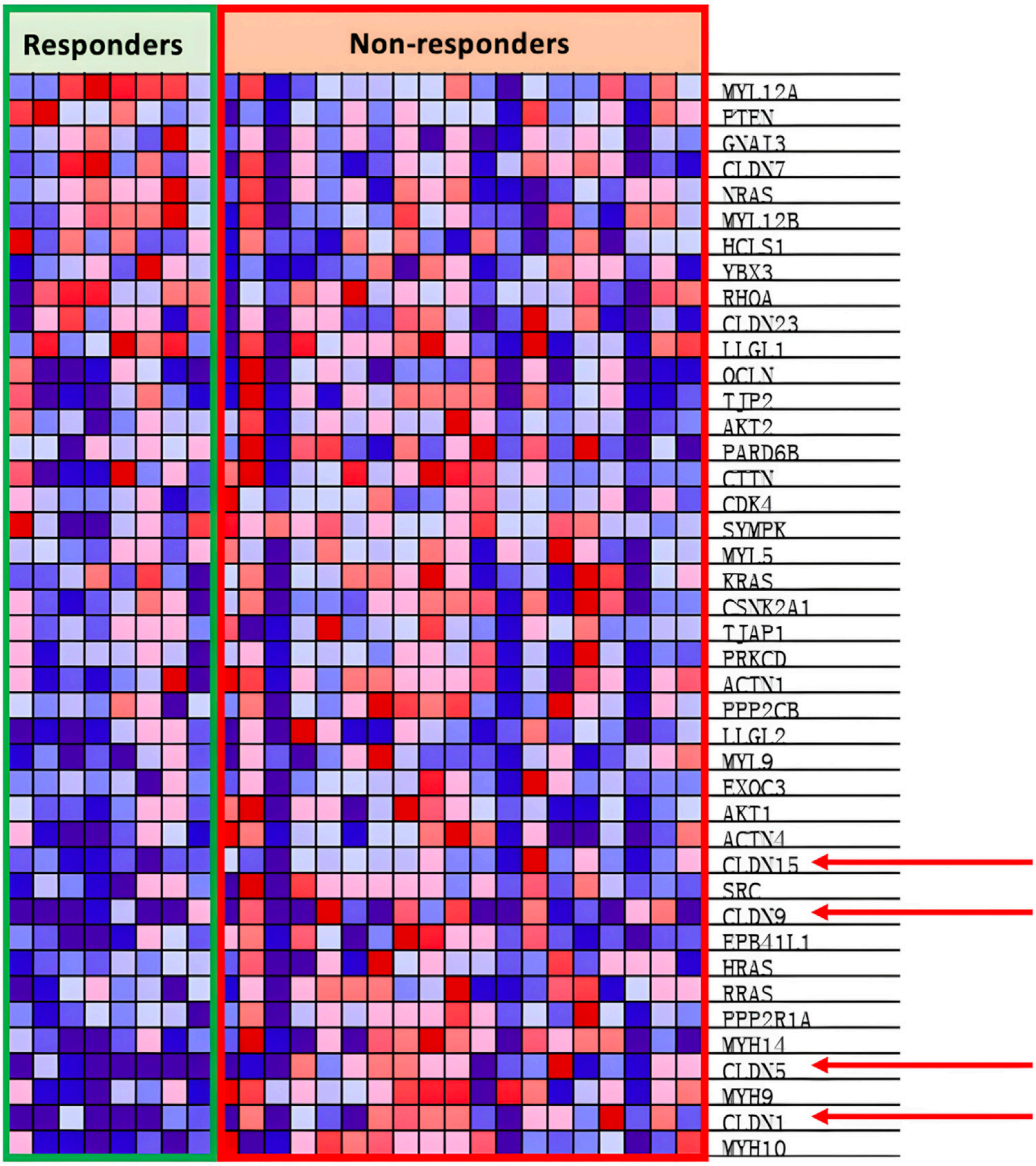
Heat map of KEGG tight junction pathway.

As shown through Figure 5, the KEGG_TIGHT_JUNCTION pathway is positively correlated with non-responsive patients.

In Figure 5, the heat map shows the overexpression of the CLDN family in non-responsive patients. CLDNs are chosen out of the over-expressed genes as the family are generally tetraspan transmembrane proteins of tight junctions, which allows them to be easily identified thanks to their extracellular domain (Krause et al., 2008). Out of the CLDNs, CLDN1 is chosen as it is widespread within the lung epithelia and endothelia as seen in Figure 5. Furthermore, prior research has shown that CLDN1 promotes drug resistance of NSCLC to cisplatin, showing how CLDN1 is correlated with poor prognosis of NSCLC (Fagerberg et al., 2014). The addition of CLDN1 into the viral genome would result in the release of CLDN1 as cancer antigens upon oncolysis, encouraging cancer recognition as CLDN1 is an easily identifiable surface protein. However, research has also shown that CLDN1 could exert tumor promoter characteristics by increasing the invasion or motility of cancer cells, which could decrease the efficacy of our cancer vaccine (Chao et al., 2009; Sun et al., 2016; Bhat et al., 2020). Experiments from Hutzler et al. (2017) show increased antigen-specific immunity and anti-CLDN6 antibody production when the B16-F10 melanoma cell line was treated with recombinant measles virus-encoded with the CLDN6 gene. In summary, the addition of CLDN1 into the viral genome would increase the immunorecognition of NSCLC cells; however, it may also promote tumor metastasis if the amount is not attenuated.

CLDN1 will act as the main sensitizing antigen in this OV. Hence, the function required of CLDN1 is to be the ligand for HLA1 and HLA2 recognition. However, the full length of the CLDN1 gene spans 633bps. To shorten this gene, only the epitope region will be introduced to the viral genome.

**FIGURE 6:**
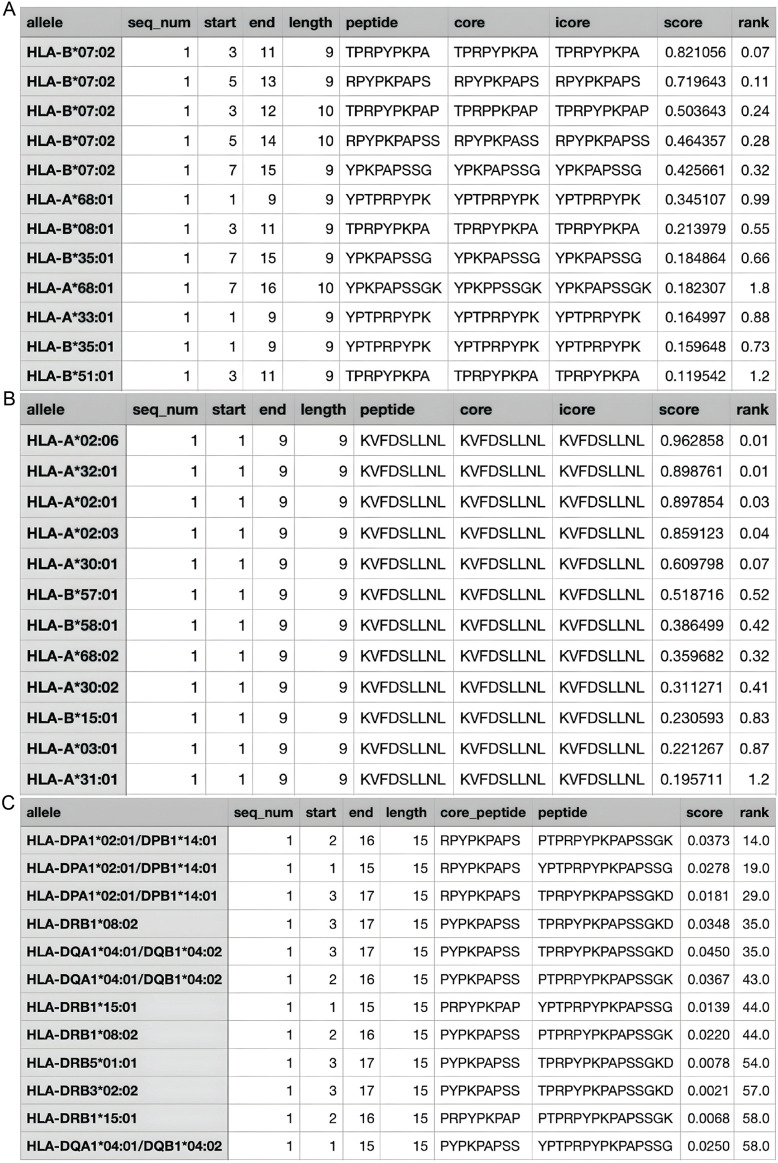
Estimated Values for HLA Affinity. **(A)** The predicted affinity of CLDN1 epitope YPT to HLA1; **(B)** The predicted affinity of CLDN1 epitope KVF to HLA1; **(C)** The predicted affinity of CLDN1 epitope YPT to HLA2. The greater the “score” value, the higher the affinity predicted, and the higher the “rank”—a categorization of the predicted affinity to a relative percentile.

According to the Immune Epitope Database (IEDB), there are two main epitope regions on CLDN1: YPTPRPYPKPAPSSGKD (YPT) and KVFDSLLNL (KVF) (Vita et al., 2018). YPT has affinity for both HLA1 and HLA2 receptors while KVF only has affinity for HLA1 (Goncalves et al., 2021; Marcu et al., 2021). However, as seen on Figures 6A–C, when running the IEDB MHC binding prediction algorithm, KVF showed a much greater affinity for HLA1 than YPT. Additionally, HLA1 is expressed on a wider range of cells than HLA2. Hence, in interest of immune activation, the epitope region KVF was chosen to be inserted into the viral genome. The KVF epitope is 27 bp in length.

#### 4.3.2 Expression of IFN-gamma

**FIGURE 7:**
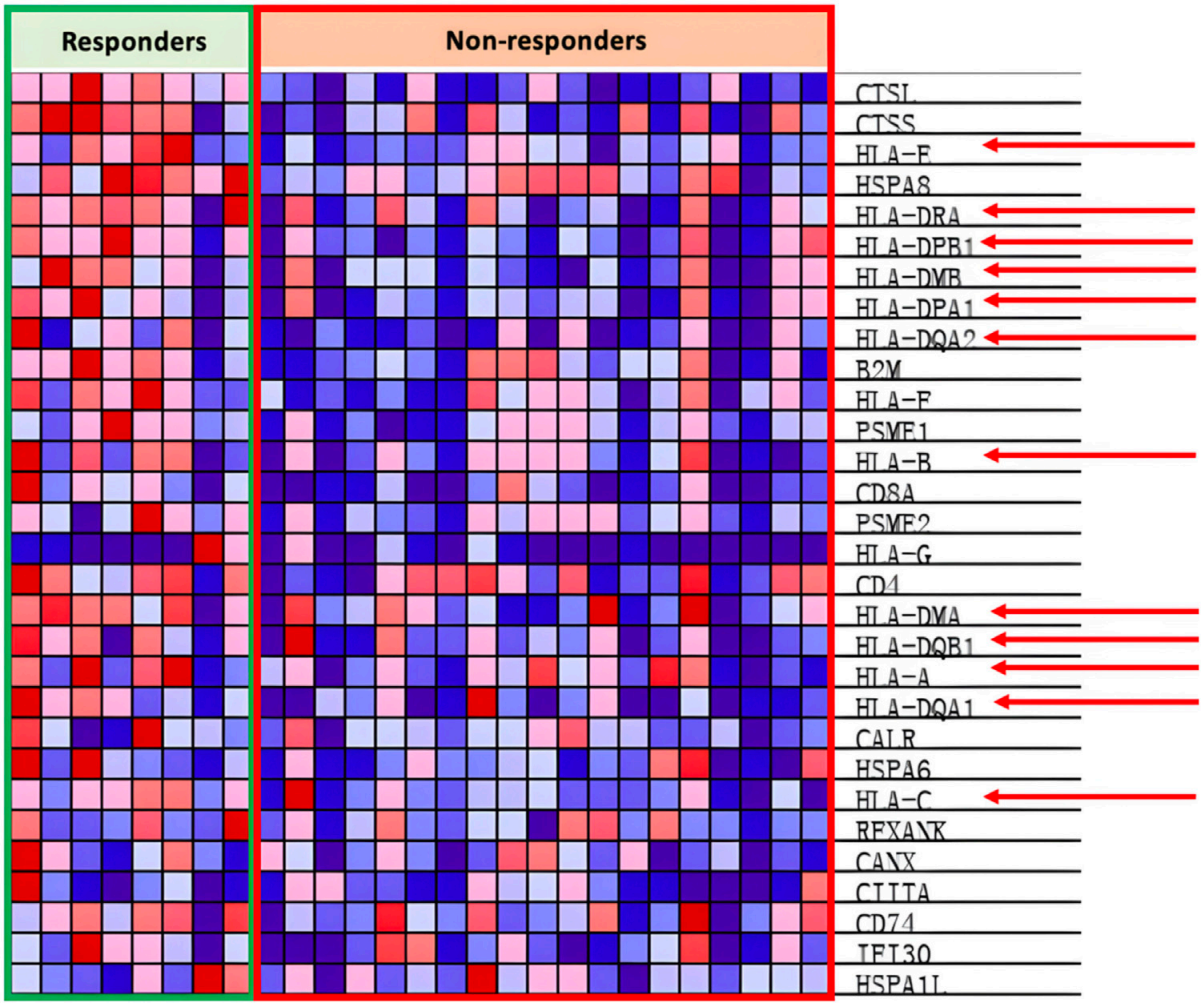
Heat map of KEGG antigen presentation pathway.

In the antigen presentation pathway, which is enriched in the PR group as shown in Figure 4B, the HLA family of proteins is under expressed within the PD group as seen in Figure 7.

HLA proteins, also known as MHCs, are vital in the process of immunorecognition and antigen presentation. Hence, one of the methods of improving response could be to increase MHC presence and stimulation. A prime candidate is interferon-gamma (IFN-gamma), a chemokine that has shown capabilities of restoring HLA to HLA-deficient lung cancer (Traversari et al., 1997). Furthermore, research has already shown that certain oncolytic viruses, such as the vaccinia virus, are capable of inducing IFN-gamma synthesis and secretion within cancer cells and have shown potent tumor regression potential (L. Chen et al., 2021; Li et al., 2022). Samson et al. (2018) conducted an *ex-vitro* experiment treating high-grade glioma (HGG) cells with reoviruses encoded with IFN-gamma, showing increased PD-L1 expression to be strongly upregulated by IFN-gamma, which benefits future anti-PD-L1 treatment (Li et al., 2022).However, IFN-gamma also activates anti-viral mechanisms with its signaling, resulting in viral degradation in the immune environment, potentially decreasing the anti-tumor efficacy of the OV (Kang et al., 2018; Li et al., 2022). Furthermore, Song et al. (2019) reported that although high doses of IFN-gamma stimulate the classical JAK/STAT pathway, low doses (0.1 ng/mL) of IFN-gamma induce activation of ICAM1-PI3k-Akt-Notch1 signaling in cancer cells, leading to increased cancer cell stemness and CD133 expression (Jorgovanovic et al., 2020). The IFN-gamma induced cancer stemness facilitates NSCLC metastatic growth, and the upregulation of CD133 tumor cells is positively correlated with poor prognosis within NSCLC patients (Song et al., 2019). Hence, although IFN-gamma has the potential to increase tumor regression and anti-PD-L1 treatment efficacy, drawbacks warn about the potential anti-viral and tumor-enhancing side effects of IFN-gamma relating to dosage.

The full IFN-gamma gene is 591 bp long (Pruitt et al., 2009). In this original sequence, there is a signal peptide which is usually cleaved in order for the protein to be secreted and function normally (Wang et al., 2014). In our instance, this cleavage is unnecessary as all intracellular materials will be released upon oncolysis of the cell (LaRocca and Warner, 2018). Hence, to not only decrease the length of the gene sequence but also to ensure the functionality of the protein, the signal peptide will be omitted from the transgene.

**FIGURE 8:**
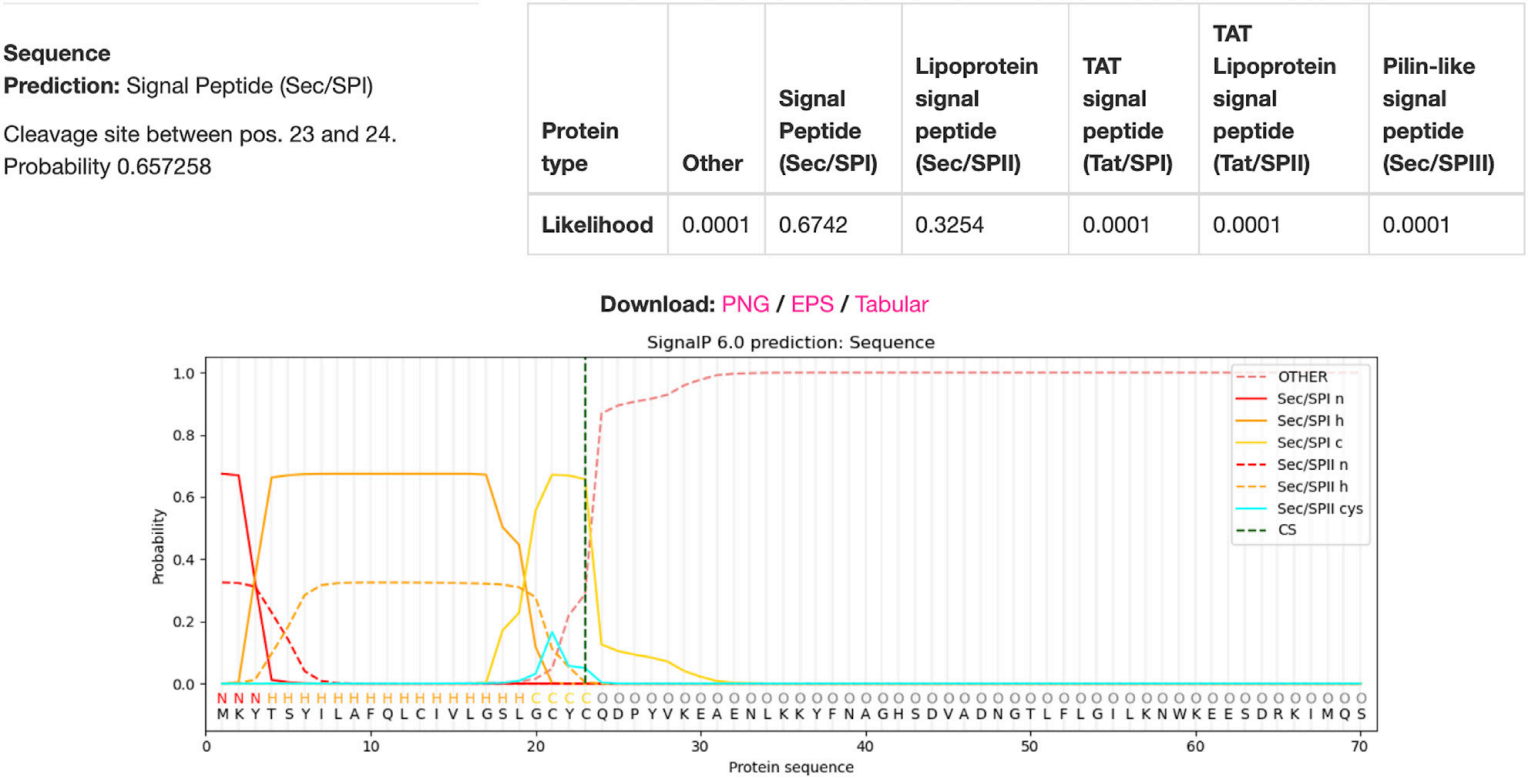
IFN-gamma Signal Peptide Cleavage Site Prediction. DTU Health Tech’s SignalP algorithm predicted that there is a 67.42% chance that there was a signal peptide cleavage site between amino acids 23 and 24.

Using DTU Health Tech’s signal peptide predicting algorithm SignaIP to predict the signal peptide cleavage site on the IFN-gamma protein, the results in Figure 8 were produced (Teufel et al., 2022). The algorithm predicted that the cleavage site would be between amino acids 23 and 24, meaning that amino acids 1-23 were part of the signal peptide complex. Hence, the remaining 522bps would be included in the viral genome whilst the 69bps that coded for the signaling peptide will be excluded.

#### 4.3.3 Expression of IL7

**FIGURE 9:**
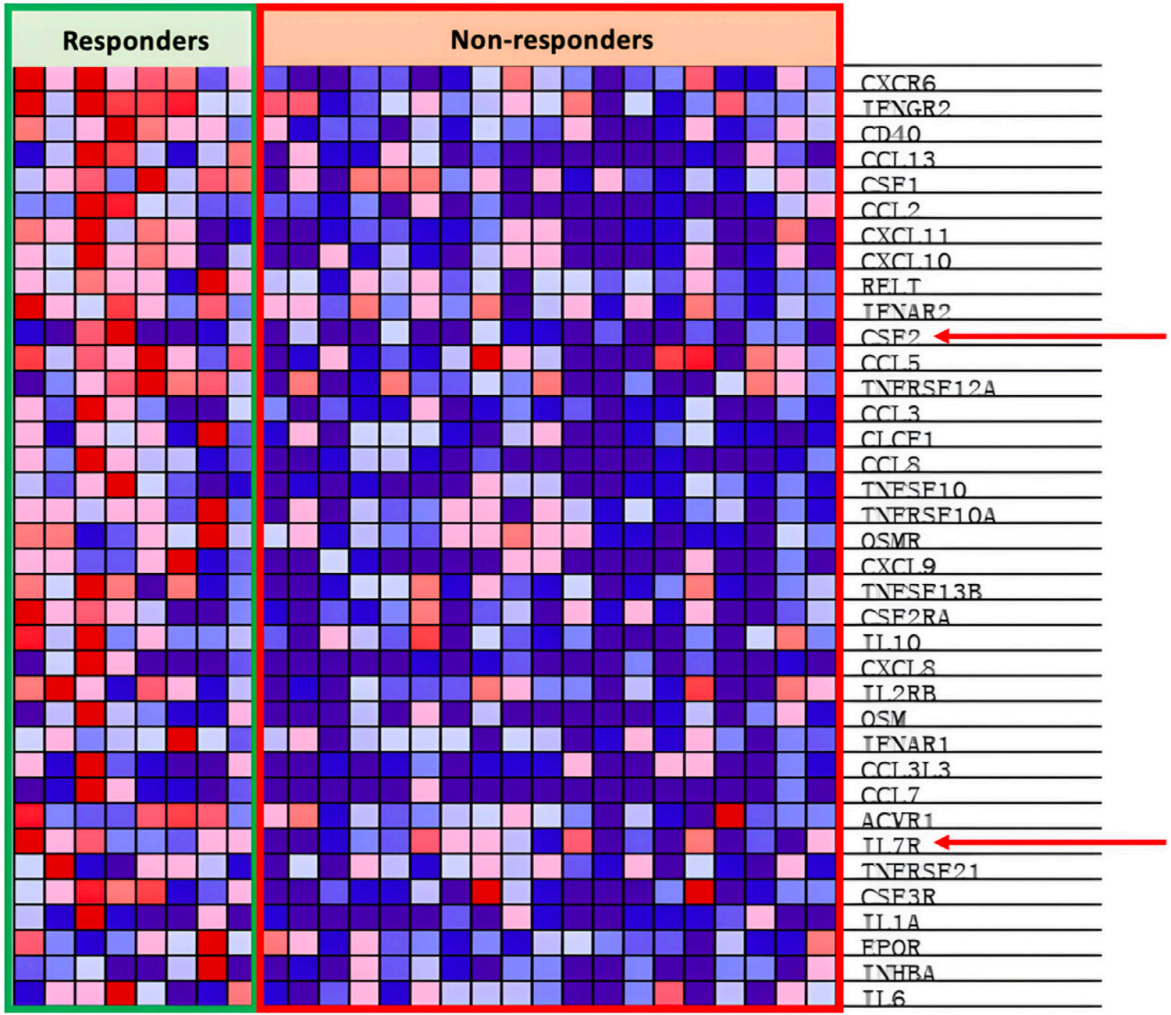
Heat map of cytokine cytokine-receptor pathway.

Interleukin seven receptor (IL7R), part of the cytokine cytokine-receptor pathway, is under expressed within the PD groups as seen in Figure 9. IL-7 stimulates anti-tumor responses such as autophagy, migration, proliferation, and angiogenesis (Lin et al., 2017; Zhu et al., 2022). IL-7 is also potent at sensitizing the TME to ICIs and antagonizing the immunosuppressive network, both of which subsequently support the goal to increase response rates to anti-PD-1 treatment (Pellegrini et al., 2009; Ke et al., 2019; Nakao et al., 2020). Nakao et al. (2020) locally injected oncolytic vaccinia viruses with the dual expression of IL-7 and IL-12 into several different types of carcinomas alongside the combinational therapy of ICIs. The results showed that there was an increased presence of tumor-infiltrating lymphocytes (TILs) and increased anti-PD-1 and anti-CTLA4 sensitivity within the injected tumor (Nakao et al., 2020). Similar effects are seen in Kudling et al. (2022) IL-7 expressing oncolytic adenovirus 5, promoting tumor regression, activating CD4^+^ and CD8^+^ T cells, and encouraging T cell migration in various cell lines of different cancers. Additionally, Shi et al. (2019) concluded in their experiment that IL-7 promotes the sensitivity of NSCLC cells to cisplatin treatment, opening possibilities of combinational treatment with chemotherapy. Hence, expression of IL-7 in oncolytic viruses shows great potential to induce anti-tumor effects individually and in combination with ICI therapy and chemotherapy.

The IL-7 cytokine is a similar case to IFN-gamma where the full genomic sequence includes a signal peptide which needs to be cleaved for proper function. The original IL-7 gene is 177 amino acids in length (531bps) (Paysan-Lafosse et al., 2022). The signal peptide cleavage site is between amino acids 26 and 27, meaning that amino acids 1-26 code for the signal peptide and amino acids 27-177 code for the functioning IL-7 protein (Ivica et al., 2021). Hence, excluding the 78bps that code for the signal peptide, a nucleotide sequence of 453bps coding for IL-7 will be included in our viral genome.

#### 4.3.4 Expression of GMCSF

Granulocyte-Macrophage Colony-stimulating factor (GM-CSF), encoded by the PR enriched CSF2 gene as seen in Figure 8, is part of the cytokine cytokine-receptor pathway. GM-CSF, as introduced earlier in the article, is vital in the differentiation and proliferation of hematopoietic cells, increasing the presence of neutrophils, effector T-cells, and APCs, leading to increased tumor regression (Ebner et al., 2003; Kumar et al., 2022). As seen by the example of T-VEC, oncolytic viruses can be armed with GM-CSF with the intention of increasing the local anti-tumor immune response. Rangsitratkul et al. (2022) inserted the GM-CSF transgene into oncolytic VSVd51 variants (an attenuated strand of vesicular stomatitis virus) in an experiment to treat bladder cancer. Results from Rangsitratkul et al. (2022) research showed that the GMCSF carrying the OV was able to enhance activation of the innate and adaptive immune system and subsequently improve survival in mice models with bladder cancer (C57Bl/6-MB49). Similarly, Malhotra et al. (2007) compared the effects of oncolytic HSVs with (NV1034) and without (NV1023) the GM-CSF transgene when treating colorectal carcinoma and hepatoma. The NV1034 variant showed significantly better anti-tumor effects compared to NV1023 under normal circumstances; however, in mice depleted of CD4^+^ and CD8^+^ T cells, no difference in antitumor effects between the two variants was observed (Malhotra et al., 2007). Hence, the GM-CSF transgene is a prime candidate to be included in an oncolytic virus due to its capabilities of increasing immune cell presence within the TME. However, it should be considered that alongside the increased presence of anti-tumor immune cells, GM-CSF simultaneously increases the presence of myeloid-derived suppressor cells (MDSCs), T-regulator (Treg) cells, M2 macrophages, and other immunosuppressive cells (Kumar et al., 2022).

The GM-CSF protein has one singular domain and is of a compact globular structure. This protein does not have a signaling peptide upon translation and all amino acid sequences are required for the functioning of the protein (Kurzrock and Dranoff, 2003). Correspondingly, the CSF2 gene which codes for the GM-CSF protein is only 432bps in length (Miyatake et al., 1985; The UniProt Consortium, 2023). Hence, the CSF2 gene does not need to be shortened or manipulated and can be introduced into the viral genome due to it being short and not having any unnecessary parts.

#### 4.3.5 Other modifications

Additional to the strategies mentioned above, research was also conducted to find more targets and methods based on recent studies in the field. These targets and methods are seen in Table 7.

**TABLE 7:** Additional potential targets of oncolytic virus editing.

Target	Rationale for target	Method	Rationale for method
Decrease Neutrophil to Leukocyte Ratio	Higher neutrophil to leukocyte ratio is associated with inferior overall survival (OS) and progression-free survival (PFS) in anti-PD1 treated patients (Bagley et al., 2017)	Insertion of CXCL13 transgene	CXCL13 has been shown to act as a chemoattractant to CD8^+^ and CD4^+^ T cells, and B cells in NSCLC, hence able to increase leukocyte presence in the area of secretion (Guo et al., 2017)
		Co-therapy with LXY2 peptide	Cyclic peptide LXY2 pharmaceutically blocks and inhibits α3β1-integrin, which accomplishes the target of diminishing neutrophil infiltration (Goldsmith et al., 1998; Liu et al., 2003)
		Insertion of IL12 transgene	IL-12 increases IFN-gamma production from NK and T cells, while simultaneously enhancing their lytic activity and increasing the efficacy of anti-PD-L1 treatment (Cassady et al., 1998; Egea et al., 2010; Harrington et al., 2019)
Increase NK Cell Infiltration	High level of NK cells in is correlated with greater PFS when associated with anti-PD-1 treatment in NSCLC (Prat et al., 2017)	Insertion of MDC transgene	Macrophage-derived chemokine (MDC) induces the direct migration of IL-2-activated NK cells (Kumar et al., 2022)
		Insertion of IL-15 transgene	IL-15 stimulates the proliferation, activation, and expansion of NK cells (Byrd and Flynn, 2014; Bitter et al., 2020) and promotes NK cell to produces IFN-gamma (Deng et al., 2017)
Decrease MDSCs	MDSCs support tumor growth and are correlated with poor response to anti-PD-1 therapy in NSCLC (Chow et al., 2019; Limagne et al., 2019; Yang et al., 2020; Kohli et al., 2022)	Co-therapy with G31P Protein	G31P is an analog of CXCL8 which has antagonistic effects against CXCR1/2 (Buller et al., 1988). The inhibition of CXCR2 signaling has been shown to decrease MDSC infiltration, suppression of angiogenesis, reduce tumor growth, and increased anti-PD-1 treatment sensitivity (Buller et al., 1988; Colamonici et al., 1995; Sigismund et al., 2018; Inoue et al., 2021)
Increase CXCR3 Signaling	The intratumoral activity of CXCR3 signaling is required for the efficacy of anti-PD-1 therapy (Chow et al., 2019)	Insertion of CXCL10 transgene	CXCL10 is a ligand of CXCR3 and hence can activate CXCR3 signaling pathways (Inoue et al., 2021)

## 5 Other concerns and future directions

OVs can specifically infect cancer cells and induce the production of transgene proteins, allowing for a controlled and predictable method of regressing tumors. However, despite the various advantages, OVs still have prominent limitations including antiviral immunity, systemic delivery, and dosing strategies. While being able to initiate anti-tumor immunity, the presence of the OV can also evoke anti-viral immunity, which can harm the OV and decrease the efficacy of OV treatment. Furthermore, certain immune signaling pathways overlap in anti-tumor and anti-viral activity, meaning that when considering which transgenes to insert into the OV, anti-tumor efficacy and viral tolerance need to be considered simultaneously. Most modern OVs are administered intralesionally and subcutaneously to ensure the infection of the virus to the local tumor. However, these methods of administration only stimulate local anti-tumor responses and are incapable of activating a systemic reaction. Intravenous (IV) injections of OVs have been considered in order to favor systemic responses to OVs, however, OVs delivered through IV injection face the difficulties of host anti-viral responses and increase the risk of host cell infection. Hence, OVs are still limited when considering the possibility of becoming a systemic treatment. The dosage of OVs per use is also another contention of limitation. While on one side, the dosage cannot be too low or else there will not be enough virions created to sustain infection; however, on the other, if the dosage is too high, the OV may further provoke inflammatory responses or even regain pathogenicity from mutations. Furthermore, certain transgene proteins require dosage control, similar to how IFN-gamma can stimulate anti-tumor responses in high doses but induce pro-tumor effects in low doses. Overall, although OVs are promising, and many of them are being approved by the FDA such as T-VEC, Oncorine, and RIGVIR, there are still many limitations regarding the survival and efficacy of the OV to be worked out and improved upon.

Combinational therapy between oncolytic virotherapy and immune checkpoint inhibitors shows great promise in increasing OS and PFS for cancer patients. The complementary nature of these therapies shows how OVs can sensitize the TME to ICIs, whether by increasing the expression of the immune checkpoints on the tumor or by increasing lymphocyte presence, resulting in the generation of a greater response to said ICI therapy. In this study, a theoretical vaccinia virus OV with capabilities of increasing NSCLC patient response to anti-PD-1 ICI therapy was presented. Further research would be aimed towards producing and testing this OV *in vitro* on cell lines to observe potential efficacies and defects and test the accuracy of predictions generated in this study.
